# English as a foreign language teacher engagement with culturally responsive teaching in rural schools: Insights from China

**DOI:** 10.3389/fpsyg.2022.990363

**Published:** 2022-09-02

**Authors:** Delin Kong, Min Zou, Jiaoyue Chen

**Affiliations:** ^1^School of Foreign Languages, Huazhong University of Science and Technology, Wuhan, China; ^2^School of Foreign Languages, Beijing Institute of Technology, Beijing, China

**Keywords:** diversity, language teacher engagement, culturally responsive teaching, rural schools, internal and external factors

## Abstract

Culturally responsive teaching (CRT) has been found to promote student engagement and enhance learning in the classroom. As an effective pedagogy, the past decade has witnessed a soaring interest in exploring teachers’ competence, self-efficacy, and influencing factors in implementing CRT across school subjects. However, scant attention has been directed to language teachers’ engagement with CRT. Given the increasing diversity in students’ socio-economic status, cultural backgrounds, learning needs and preferences in English language classrooms, CRT has also become a prominent concern in China. This study sets out to explore English as a Foreign Language (EFL) teacher engagement with CRT in rural schools in China. With a multi-case study of eight EFL teachers, the researchers collected data through individual interviews and classroom observations. Four types of teacher engagement based on the foci (i.e., knowledge and practice) were identified and were further characterized by cognitive, emotional, and social aspects. This study also taps into the internal and external factors influencing the teachers’ engagement with CRT. Implications and suggestions were provided to tackle the problems of English Language Education in rural China and contexts alike worldwide.

## Introduction

Culturally responsive teaching (CRT) refers to practices designed to build on students’ cultural, historical, and linguistic backgrounds as teaching and learning occur (Ladson-Billings, 1994; Cruz et al., 2020). Previous research has evidenced its positive role in improving student engagement, thus enhancing their learning (e.g., Gay, 2000, 2015), especially in rural schools, which generally lack access to new technologies, with students from diverse cultural and learning backgrounds (e.g., Curtis, 2021; Kumi-Yeboah et al., 2021). In China, CRT has also become an increasingly important concern in rural schools (Kong, 2021). Along with the policies to support education in rural areas in recent decades, such as *Targeted Poverty Alleviation and Rural Revitalization* (Chinese Central Government, 2018a,b), more and more city-born teachers go to work in rural schools in economically disadvantaged areas with a heterogeneous body of students (Kong and Zou, 2022). Against such a backdrop, CRT is suggested to enable teachers in rural schools to encourage students’ engagement and enhance their learning (Zhang and Wang, 2016).

The past decade has witnessed a burgeoning interest in CRT with a focus on teachers’ self-efficacy, especially in disciplines like mathematics, sciences, early childhood education, and music, and most studies were taken out in the western context (e.g., Warren, 2018; Abril and Robinson, 2019; Brown et al., 2019; Kranski and Steed, 2022). Sufficient attention has yet to be paid to language teacher engagement with CRT, not to mention the research in oriental educational contexts. Specifically, little is known about how and to what extent language teachers actively plan and teach by drawing on students’ cultural resources to support their learning in Asian countries (Zou et al., 2021). To fill this void, we aim to explore english as a foreign language (EFL) teacher engagement with CRT in rural schools in China. Due to the influence of the COVID-19 pandemic and the associated restrictions on participant recruitment and data collection, we conducted a pilot study and investigated eight EFL teachers’ engagement with CRT in rural schools in China. Despite the reduced number of participants and data sources, the findings are expected to add insightful understanding to this meaningful yet underexplored topic of language teachers’ engagement with CRT, provide first-hand information on the professional lives of this significant yet largely invisible teaching group (Curtis, 2021), and generate practical implications for language teacher education, both pre-service and in-service (Jackson and Boutte, 2018).

## Literature review

### Understanding culturally responsive teaching

Culturally responsive teaching is a teaching method and mindset that utilizes “the cultural knowledge, prior experiences, frames of reference, and performance styles of ethnically diverse students to make learning encounters more relevant to and effective for them” (Gay, 2010). It is grounded in social justice and equity with the three basic tenets of academic success, cultural competence, and social-political consciousness (Ladson-Billings, 2009).

Developing along with the cultural difference ideology in education (Ladson-Billings, 1995), CRT critiques and offers an alternative to the traditional teaching practices that have long overlooked the social, cultural, and ethnic parameters in teaching and learning (Au, 1993; Delpit, 2006). CRT, in this way, challenges the one-size-fits-all approach in traditional teaching models and places students’ experience, knowledge, and the social-historical-political context at the center of all teaching practices (Dickson et al., 2016). CRT thus intends to engage culturally and linguistically diverse students in meaningful learning activities. It seeks to empower students by maintaining their cultural integrity and making them feel pride in their cultural heritage (Hill and Torres, 2010). As Gay (2010) stressed, the essential premise of CRT is that students are more likely to achieve academic success when knowledge and skills are presented in a way more congruent with their cultural frame of reference. Teachers, therefore, are expected to spend more time learning, understanding, and integrating students’ cultural backgrounds into the content of their teaching. Moreover, teachers are encouraged to engage with CRT, upon which radical changes can be realized in their instructional processes (Gay, 2018).

Siwatu (2007) proposed that teachers can implement CRT in four essential aspects, i.e., curriculum and instruction, classroom management, student assessment, and cultural enrichment. Firstly, in *curriculum and instruction*, teachers can utilize students’ cultural knowledge, experiences, prior knowledge, and individual learning preferences to design culturally relevant curricula and classroom activities to meet students’ affective, cognitive, and educational needs and enhance the reciprocal process of teaching and learning. Secondly, regarding *classroom management*, teachers can incorporate students’ cultural orientations to create a culturally compatible classroom environment that is warm, comfortable, and supportive to minimize the effect of cultural mismatch. Teachers thus can effectively communicate with students and develop a community for learners. Thirdly, in *student assessment*, teachers can provide multiple opportunities, use various techniques to assess students’ learning, and interpret students’ performance in standardized tests based on their knowledge about students. Lastly, as to *cultural enrichment*, teachers can provide students with the necessary knowledge and skills to navigate the mainstream culture without sacrificing their cultural identities, native languages, and connections to their own cultures.

As one of the most influential studies in CRT, Siwatu’s framework has been widely used in the area (e.g., Chahar Mahali and Sevigny, 2021; Chu, 2022; Ciampa, 2022), whereby its validity and reliability have been repeatedly examined and ensured (Dickson et al., 2016). Therefore, this framework was adopted to develop the data collection instruments (i.e., interview protocol and classroom observation sheet) and the priori coding scheme for data analysis.

### Culturally responsive teaching and English language education

In English language education, CRT has become a buzzword in recent decades. Previous research primarily focused on CRT in English as a first language (L1) education and English as a second language (L2) education (Aronson and Laughter, 2016) and has evinced the positive correlation between CRT and students’ engagement and English learning motivation (Hill, 2012).

Existing studies on CRT in L1 education were mainly conducted in urban schools in the United States with multi-ethnic students (e.g., African American, Latino, and Asian) and/or white students from low social-economic-status families. The main research foci were choosing culturally relevant teaching materials, trying CRT-oriented teaching approaches, documenting teachers’ CRT experiences and identifying possible influencing factors over teachers’ CRT practices. For example, regarding choosing culturally relevant teaching materials, Christianakis (2011) examined the effectiveness of rap and poetry in facilitating urban fifth-grade students’ engagement with English learning. She found that such materials aroused students’ interest and motivated them to finish assignments. Concerning trying CRT-oriented teaching approaches, Iwai (2019) probed using multicultural literature genres to realize CRT and found positive impacts on teachers, including increased awareness, developed knowledge, and improved skills in practicing CRT. Keehne and her colleagues proposed a five-element indigenous framework for CRT-oriented literacy teaching, including (a) literacy in indigenous languages; (b) community connections; (c) a shared vision that encompasses culture, academic proficiency, and community; (d) authentic assessment; and (e) teaching grounded in culture and higher-level thinking (Keehne et al., 2018). Lastly, in documenting teachers’ experiences and identifying possible influencing factors, Coffey and Farinde-Wu (2016) observed a black female English teacher conducting CRT. They found that teachers’ interconnectedness with their students regarding their cultural backgrounds can influence the effectiveness of CRT practice. Valtierra and Whitaker (2021) also looked into an English teacher’s CRT experiences and found that the compatibility between teachers’ CRT beliefs and the teaching contexts can influence their practices.

Compared with CRT in L1 education, research on CRT in L2 education is relatively scarce and existing studies are also primarily conducted in the United States with immigrants. Previous research has examined L2 teachers’ self-efficacy in CRT and identified critical influencing factors over L2 teachers’ CRT practices. Siwatu and his colleagues examined eight pre-service L2 teachers’ CRT self-efficacy. They found that pre-service L2 teachers recognized the value and utility of CRT practices but generally doubted their ability to conduct CRT due to the lack of related knowledge and skills (Siwatu et al., 2016). Various influencing factors have been found in the previous studies on CRT in L2 education, including teachers’ understanding of students’ cultural backgrounds and their eagerness to integrate such knowledge into their teaching content (Nieto, 2010), and L2 teachers’ high expectations and strong confidence in students’ ability (Lee, 2010).

The existing studies have provided valuable insights about CRT in language education. However, compared with other disciplines (e.g., maths, science, early childhood education), research in this area still remains insufficient and inconclusive. Few studies have systematically examined language teachers’ engagement with CRT. Therefore, little is known about the approaches and the extent that language teachers would proactively enact students’ cultural resources to support their learning (Zou et al., 2021). Moreover, given that most existing studies were conducted in the western context (i.e., United States), oriental insights are needed to contribute to a more well-round panorama of CRT research in language education.

### Teacher engagement and English language education

Teacher engagement reflects “the voluntary allocation of physical, cognitive, and emotional resources across teaching-related activities” (Perera et al., 2021, p. 883). Klassen et al. (2013) indicated that teacher engagement is a multidimensional construct composed of cognitive, emotional, and social engagement (with students and colleagues). *Cognitive engagement* denotes how teachers concentrate on, invest their energy in, and utilize their mental resources in their work; *emotional engagement* refers to teachers’ feelings about, degrees of devotion to, and emotional response to their work; *social engagement* with students and colleagues denotes teachers’ energy dedicated to connecting and interacting with students and colleagues. Over the past decade, Klassen et al.’s (2013) framework of teacher engagement has been employed in studies across different contexts (e.g., Granziera and Perera, 2019; Wu et al., 2022). In particular, Ho et al. (2021, p. 5) validated this framework in China and concluded that teacher engagement could be represented as “a single superordinate construct” with the three underlying dimensions.

In language education, teacher engagement has gradually gathered more academic attention. While language teacher engagement with CRT is still underexplored, existing studies on language teacher engagement with other teaching and learning activities can offer useful references, mainly regarding the forms of engagement and influencing factors. Regarding the forms of engagement, Jiang et al. (2021) explored language teacher engagement with digital multimodal composing and identified three forms of engagement, namely, incidental, ambivalent, and integral. Zou et al. (2021) explored language teacher engagement with online formative assessment in EFL writing during COVID-19 and found three forms: disturbing, auxiliary, and integral. Previous studies have also revealed that internal and external factors influence language teacher engagement with various teaching and learning activities. Internal factors include teachers’ beliefs, motives, characteristics, and physiological and emotional states. External factors contain working contexts and climates, pedagogical theories, national guidelines on EFL teaching, teaching norms of the schools, prescribed curriculum, and high-stakes testing regimes (e.g., Bao et al., 2021; Jiang et al., 2021; Wu et al., 2022).

Building on the insights of previous works, this study aims to systematically explore EFL teacher engagement with CRT in rural schools in China. Two research questions are proposed:

1) How do EFL teachers engage with CRT in rural schools?
2) What internal and external factors have influenced EFL teachers’ engagement with CRT in rural schools?

## Methodology

A qualitative case study design was adopted to answer the two research questions. This study was situated in three rural schools in central China, an important area in the Chinese educational reform (Gu, 2010). Participants were eight EFL teachers who worked in the three rural schools. They were recruited by convenient sampling. During the summer vacation of 2020, the corresponding author contacted an officer in the local education bureau. The officer acted as a gatekeeper, helped contact all the EFL teachers in three rural schools, and briefly introduced this study. The corresponding author then sent a short message to each teacher to explain this study in detail and queried their willingness to participate. Ten teachers responded positively, but two quit before this study. One teacher was pregnant, and the other was temporarily assigned to teach Chinese instead of English in the coming semester. Thus, we had eight teachers as the final participants. They were all female teachers and pseudo-named Cuiju, Dujuan, Fengxian, Meigui, Qiangwei, Shancha, Shaoyao, and Shuixian. Table 1 displays their demographic information. As shown in Table 1, all the eight EFL teachers share very similar educational background. Although they vary in years of teaching experience, they are all new to the rural schools as they have less than 2 years of teaching experience in those schools.

**TABLE 1:** Participants’ profiles.

Name	Gender	Total years of teaching; Years of teaching in rural schools	Educational background
Cuiju	Female	0.5 year; 0.5 year	BA (Translation)
Dujuan	Female	12 years; 1 year	MA (English Teaching)
Fengxian	Female	0.5 year; 0.5 year	BA (English for International Business)
Meigui	Female	1 year; 1 year	MA (Translation);
Qiangwei	Female	14 years; 1 year	BA (English Teaching)
Shancha	Female	2 years; 1 year	BA (English Teaching)
Shaoyao	Female	3 years; 1.5 years	BA (English for International Business)
Shuixian	Female	3 years; 1 year	BA (English Language)

Data were collected from semi-structured interviews and classroom observations. Meanwhile, participants were invited to share their lesson plans and PowerPoint files for teaching as complementary data on a voluntary basis. The interview and classroom observation protocols were designed based on previous teacher engagement and CRT research. Specifically, in interviews, teachers were invited to talk about their CRT practices in the four aspects of curriculum and instruction, student assessment, classroom management, and cultural enrichment. Questions asked were derived from items in Culturally Responsive Teaching Self-Efficacy (CRTSE) (Siwatu, 2007), such as “Do you do needs analyses? If yes, why and how do you do it? If no, why?,” “Do you assess students in different ways? If yes, why and how do you do it? If no, why?,” “Do you pay attention to including every student in your teaching? If yes, why and how do you do it? If no, why?,” and “Do you critically examine the curriculum and textbook content to determine whether it reinforces negative cultural stereotypes? If yes, why and how do you do it? If no, why?”. Toward the end of each aspect and of the interview, teachers were invited to utter their cognitive, emotional, and social engagement with realizing CRT in the specific aspect and their teaching in general (Klassen et al., 2013). In classroom observations, the researchers took detailed notes regarding the four aspects of CRT and three dimensions of teacher engagement. The researchers planned to observe five periods of each participant but were only able to observe one for each due to the COVID-19 pandemic and the strict preventive measures of the schools. The interviews were conducted individually with each participant, lasting about 2 h. All the interviews were done in Chinese, the participants’ first language, and later transcribed and translated into English by the researchers.

A qualitative, inductive approach was employed to analyze the data (Miles et al., 2018). The researchers first read all the transcripts and worked independently to identify emerging codes. They then worked together to juxtapose and compare the identified codes. Using the Chinese concepts of “*zhi*” (which refers to knowledge) and “*xing*” (which denotes practice), the researchers identified four types of engagement among the eight participants. To further enhance the trustworthiness, all the data and interpretations were sent back to participants for member checking, and their comments were used to refine the final results. Table 2 displays a summary of the main findings of this study.

**TABLE 2:** A summary of main findings.

Participants	Teacher engagement	Cognitive	Emotional	Social
Shuixian and Meigui	*xing er bu zhi:* Teachers practise CRT well but do not know the concept well.	Low	High	High
Dujuan and Fengxian	*zhi er bu xing:* Teachers know CRT but do not practise it.	High	Low	Low
Shancha and Cuiju	*bu zhi bu xing:* Teachers neither know nor practice CRT.	Low	Low	Low
Shaoyao and Qiangwei	*zhi xing he yi:* Teachers know CRT and practise it well.	High	High	High

## Findings

This section will present the findings regarding the four types of engagement with CRT among the participants.

### *Xing er bu zhi* (行而不知): Shuixian and Meigui

Shuixian and Meigui are both young female English teachers. Shuixian was working in a rural public fund primary school at the time of this study. She took the government-sponsored teacher recruitment examination in 2021 and successfully obtained an officially budgeted teaching post^1^ (*jiaoshi bianzhi* in Chinese). Different from Shuixian, Meigui was working in a private rural secondary school. She wanted to work in a company but became an English teacher by accident in 2020. Regarding CRT, both teachers practiced the “*xing er bu zhi*” type of engagement. While they demonstrated a high level of emotional and social engagement with CRT, they did not realize that they were actually practicing CRT, thus a low level of cognitive engagement.

In terms of the curriculum and instruction, Shuixian and Meigui practiced CRT and were driven by the beliefs that “knowledge reshapes destiny” (Shuixian-Interview) and “English is important” (Meigui-Interview). In particular, Shuixian believed that English constituted an essential category in students’ knowledge repertoire for their future. However, rural students lack motivation in English language learning due to the relatively unsatisfied English teacher workforce quality, outdated English teaching methods, and insufficient emphasis from their parents. Meigui believed English was important for some professions, like doctors and scientists. However, most rural students failed to realize this because of limited or stereotyped views of English learning from teachers and parents, e.g., “only those who want to go to foreign universities need to learn English” (Meigui-Interview). Informed by such beliefs, both teachers wanted every student to develop a positive understanding of English learning and benefit from their teaching. They conducted needs analyses for their courses and learned various characteristics of rural primary school students.

*First, “interesting” is a common standard in their eyes for every subject. Second, they know little about the outside world. Third, they only study in class and seldom study after class, so class time is important and precious. Lastly, some girls have long been overlooked since their parents are patriarchal and only care about their elder/younger brothers’ studies.* (Shuixian-Interview)

To make her teaching more interesting, engaging, and effective for all the students, Shuixian tried to connect her teaching content to students’ daily life. For example, when teaching different colors in Grade 3, Shuixian did the following, and students became very excited in the class.

*Shuixian: What color is it (pointing to a boy’s coat)?*
*Students: Blue.*
*Shuixian: What color is it (pointing to the tea leaves in her cup)?*
*Students: Green.*
*Shuixian: What color is it (pointing to the national flag on the wall)?*
*Students: Red.*
*Shuixian: Only red? What color is it (pointing to the stars on the flag)?*
*Students: Yellow!*
*Shuixian: Good!*
(Shuixian-Classroom Observation)

In addition, both teachers adopted various teaching methods, including game teaching, situational language teaching, and total physical response. As explicated by Meigui, what was important in rural English teaching was to attract students’ interest and help them realize the significance of English learning. By playing games, creating real-life scenarios, and learning by doing, “students would follow the teacher intensively, enhance their learning efficiency, and become more devoted to English learning” (Meigui-Interview).

Concerning classroom management, both teachers also implemented CRT, which was underpinned by their beliefs and knowledge about students. To elaborate, Shuixian believed that “students should be the little masters of their classrooms” and learned that “most students are left-behind children and are living with grandparents who provide inadequate emotional support” (Shuixian-Interview). In her teaching, Shuixian created many opportunities for students to collaborate, cultivate their teamwork spirit, and engage in healthy competition for English learning, with an overarching aim of “progress and grow together” (Shuixian-Interview). After class, Shuixian tried to build mutual trust and affective bonds between her and the students. She chatted with them, joined their games like hawk-and-chicken, paid particular attention to their emotional status, and provided psychological counseling if necessary.

Meigui considered the identity of “a reliable teacher” beneficial in classroom management since students “prefer reliable teachers and trust them more” (Meigui-Interview). According to Meigui, a reliable teacher should have at least three qualities: egalitarian, creditable, and encouraging. To align with such expectations, Meigui often chatted with students, told them they could cry on her shoulders at any time and promised to be a secret keeper. Moreover, she always tried to fit her deeds to her words, e.g., “if I promised to give students some rewards for their good test performance, I would definitely do that” (Meigui-Interview). Lastly, in her classroom teaching, Meigui tried to interact more with those silent students and always gave positive feedback as encouragement. By doing so, Meigui wanted “every student to realize that they are important in this classroom” (Meigui-Interview).

Regarding CRT in student assessment, both teachers avoided evaluating students’ language learning achievement based solely on the paper-and-pencil tests. Though it was predominant in China that rural schools (including Shuixian’s school) compared their students’ mean scores in all subjects with urban students as an important index for their teaching quality, Shuixian thought such comparisons were discouraging and meaningless since “rural students could hardly surpass their urban counterparts in English grades, and such comparisons can only make both rural students and teachers more discouraging” (Shuixian-Interview). To Shuixian, rural students should compare with no one else but themselves. To her, the ultimate purpose of student assessment in rural English teaching was to encourage students instead of discouraging them. Therefore, teachers should adopt different assessment methods to identify students’ development. In a similar vein, Meigui emphasized the overall quality of the assessment. In her class, for example, one student could get very satisfactory marks on almost every test (more than 115 out of 120) but turned out to be a troublemaker in the class by constantly interrupting the teaching and learning process. This experience made Meigui realize that teachers should appraise students’ English learning beyond the paper-and-pencil tests to obtain an accurate and consistent assessment.

Lastly, the two teachers differed in conducting CRT in cultural enrichment. Shuixian practiced more on this, informed by her belief that “one of the ultimate goals of language teaching is helping students thrive (Shuixian-Interview). Shuixian’s CRT practices regarding this aspect had two categories. On the one hand, she taught her students manners and etiquette so they could behave more appropriately. For example, from Shuixian’s observation, most rural students lacked etiquette or manners as their parents and/or guardians did not teach them, e.g., “some students never say thank you after getting candies (as a reward) from me” (Shuixian-Interview). The job of correcting those rural students’ inappropriate behaviors and teaching them to be polite and respectful was finally left to the teachers. On the other hand, she kept reminding students of their own cultures while learning about western cultures. For instance, she integrated local cultures (e.g., tea culture in the town) and Chinese traditional cultures (e.g., Chinese Kongfu and Erhu) in her teaching content. Meanwhile, she consciously enhanced students’ sense of pride in such cultures. By comparison, Meigui did little, mainly due to her unfamiliarity with the local cultures and unawareness of emphasizing Chinese cultures in English teaching. As mentioned above, Meigui became a rural English teacher by accident and did not plan to stay in her school or even this profession for long. She was not entirely satisfied with her current working context and still regarded herself as a visitor to the school for a temporary stay.

In sum, this section displays Shuixian’s and Meigui’s engagement with CRT in their teaching, and both teachers practiced CRT well in their work. Shuixian realized CRT in all four aspects, i.e., curriculum and instruction, classroom management, student assessment, and cultural enrichment. Although Meigui did not do much in cultural enrichment, she did an excellent job in the other three aspects. However, it ought to be noted that both teachers did not realize that they were practicing CRT, and their practices were underpinned by their practical knowledge. As for the influencing factors, the two EFL teachers’ engagement with CRT was influenced by both internal and external factors. The internal factors included teachers’ beliefs, professional identity (to be a reliable and trustworthy teacher), pedagogical knowledge (applying various teaching methods) and their knowledge about the students, and commitment to the profession as an EFL teacher, which led to their engagement with CRT. However, the test-oriented culture, as one prominent external factor, put certain limitations on both teachers’ classes, as the rural students’ scores in (high-stake) exams were the benchmark for their academic success and their teachers’ performance. Appropriate pre- and in-service professional training was also recognized as an external factor. They ascribed their ignorance of the theoretical understandings of CRT to “the lack of pre-service teacher education” (Shuixian-Interview) and “the absence of in-service learning opportunities at rural schools” (Meigui-Interview).

### *Zhi er bu xing* (知而不行): Dujuan and Fengxian

Dujuan is an expert English teacher with more than 10 years of teaching experience. At the time of this study, she worked in a rural public junior secondary school. She served as the head of the English teaching panel for Grade 9, when teachers and students were busy preparing for the senior high school entrance examination (*Zhongkao*)^2^. Fengxian, by comparison, was an extremely young teacher. She was born in 2000 and had been working in a rural private junior secondary school for only about half a year. She took over a teacher’s class halfway and taught Grade 8 students. Regarding CRT, both teachers were in the “*zhi er bu xing*” type of engagement. Both teachers said they knew about CRT, acknowledged its significance and were willing to integrate CRT into their teaching. However, due to some practical concerns related to *Zhongkao*, they did not practice CRT well in their teaching, thus demonstrating a high level of cognitive and emotional engagement but a relatively low level of social engagement with different stakeholders, i.e., students, colleagues, and parents.

Concerning curriculum and instruction, both teachers acknowledged that they did not implement CRT well due to different beliefs. Dujuan believed that “in Grade 9, *Zhongkao* prioritizes everything and directs teachers’ teaching” (Dujuan-Interview). In her teaching, Dujuan had the habit of critically scrutinizing the contents presented in the textbook and relating them to rural students’ knowledge repertoire. Still, she decided to teach the content without much adaptation even if she spotted some problems and wanted to change because she worried related content might appear in the examination paper. For example, when preparing the lessons concerning the unit “It Must Belong to Carla,” she found one passage was about Stonehenge. While Dujuan immediately realized that rural students must be strange with Stonehenge, she still decided to analyze the passage line by line. That lesson, unsurprisingly, had a very dull atmosphere. After the class, Dujuan reflected:

*Most rural students know nothing about it, and I cannot understand why the editors put a passage about Stonehenge here. When planning the lesson, I once considered replacing it with passages about world heritages in China, such as the Great Wall and Terra Cotta Warriors. However, I worried Stonehenge would appear on the examination paper, so I gave up, braced myself, and analyzed the passage.* (Dujuan-Classroom observation)

Unlike Dujuan, Fengxian’s willingness to realize CRT in curriculum and instruction was hindered by her limited teaching experiences and knowledge about students. She believed “my students and I are as ignorant as white papers” (Fengxian-Interview). It seemed to be very challenging for Fengxian to match students’ knowledge, background, and learning needs with her teaching. On the one hand, she knew little about her students since she had only taught for only several months. On the other hand, based on her limited knowledge about students, she learned that students had problems uttering their knowledge, backgrounds, and learning needs and commented that “they are too young to know themselves” (Fengxian-Interview). Meanwhile, since Fengxian’s school was a boarding school, students’ parents also knew little about their children’s learning. She could not obtain much information from the parents, either. All these problems restricted Fengxian’s knowledge about students, thus a low level of engagement.

Regarding classroom management, the two teachers also did not implement CRT. Dujuan believed that “teachers must dominate in Grade 9 classrooms” (Dujuan-Interview). Dujuan acknowledged the importance of creating an engaging classroom atmosphere but said she was more willing to do that in teaching Grades 7 and 8 students. Her reasons were twofold. First, she thought that in Grade 9, as teachers and students were busy preparing for *Zhongkao*, the class time was so limited, and it was time-consuming to cater to every student’s needs and feelings. Also, Dujuan found that some students in Grade 9 were less engaged in English learning. They either believed they could get admitted by a senior high school without sparing too much effort on English, or they decided to quit studying and work after Grade 9, the last school year of compulsory education in mainland China. Dujuan once tried to involve every student but failed with negative feedback from some of them, like “you are so annoying” and “I just do not want to learn English” (Dujuan-Interview). Such comments and experiences put off Dujuan’s enthusiasm and made her reluctant to spare more effort.

Fengxian upheld the significance of CRT in classroom management and believed the saying by Confucius that “a student will believe in teachings only when he gets close to his teacher” (*qin qi shi xin qi dao*). Therefore, at the beginning of her teaching, she tried her best to be friendly and approachable to students. However, this did not work well in the rural school. Some unruly students did not listen to Fengxian and treated her as “a novice teacher and a good-tempered pushover” (Fengxian-Interview). Some other students were rebellious and refused to communicate with her. Such adverse experiences also pushed Fengxian to become more dictatorial to establish and present her authority as a teacher in the classroom. But Fengxian did not plan to be dictatorial all the time. Once her teacher authority was built, she still wanted to switch back to being a friendly and approachable teacher.

As for student assessment, while both teachers agreed that they should provide ample opportunities and assess students from different perspectives, they still demonstrated a low level of engagement in their teaching. For Dujuan, she was pushed to focus on students’ performance in standardized tests since “marks are of great importance in Grade 9” (Dujuan-Interview). At this stage, the school evaluated teachers’ work using the index of “one score and three percentages” (*yi fen san lv*), which referred to students’ mean score and the three percentages of fail, pass, and distinction in the test series for *Zhongkao*. Such a practice pushed Dujuan to emphasize students’ marks in standardized tests, though she knew that “it was unfair for some students who perform well in my class, but are not good at testing” (Dujuan-Interview).

Working in a private school, Fengxian did not have to face the “one score and three percentages”-oriented teacher evaluation. However, she was forced to focus on students’ performance on standardized tests, though she considered it inappropriate. Fengxian’s school was newly founded and had to compete with public schools for the student source. Under such circumstances, students’ marks in standardized tests were regarded as the primary indexes for the school’s teaching quality, which directly relates to its reputation and parents’ choice. The much higher tuition fees can be a concern for most families in that area (400 RMB per year for public schools, but 10,000 RMB per year for Feng Xian’s school). Therefore, once their children were enrolled at the school, the parents wanted to repay their financial investment in their children’s education, i.e., excellent academic performance or satisfying test marks in Zhongkao. As a new teacher, Fengxian had no other options but “conformed to such expectations from the school and students’ parents” (Fengxian-Interview).

Lastly, regarding CRT in cultural enrichment, both teachers agreed that it was essential to integrate cultural elements in English teaching to navigate students to learn the mainstream cultures and also preserve their own cultural identities. However, both teachers confessed that they did not practice well due to some practical concerns. For Dujuan, she thought that “cultural enrichment is a long-term enterprise and will not get effect instantly” (Dujuan-Interview). Integrating cultural elements was time-consuming, especially in busy stages like Grade 9. Though Dujuan thought cultural enrichment was necessary, as it was related to value education, her purposive ignorance of this session would not stop as long as *Zhongkao* still existed. She reflected:

*I believe most Grade 9 English teachers have a similar mindset to mine. As long as Zhongkao still kidnaps us, it is hard for us to do cultural enrichment, as it will not appear in the examination papers.* (Dujuan-Interview)

Fengxian also did not do well in cultural enrichment. Her problem was that her “teaching time was spent on language itself, but not cultures and values, due to students’ unsatisfying English proficiency” (Fengxian-Interview). Some of Fengxian’s Grade 8 students knew nothing about phonetic symbols, and some even had problems recognizing the 26 alphabetic letters. Though Fengxian realized that her teaching contents should have lower-order (language) and higher-order (cultures and values) categories, most times, she only had time to cover the former.

In sum, while both teachers acknowledged the significance of realizing CRT in teaching, they did not practice well due to both internal and external influencing factors. While both teachers were new to rural schools, Dujuan developed her ways of gaining knowledge about her students, communicating with the parents, establishing her authority in class, and using different methods in class management to improve the effectiveness of her teaching during her 10-year English teaching experience in urban schools. Meanwhile, Fengxian seemed to be a little struggling as she was still a novice teacher and still in a process of learning. Similarly, their engagement with CRT was constrained by the external factor, test-oriented culture, where the students’ scores were used for evaluating learning and teaching. It thus had created extra burdens for the EFL teachers in rural schools.

### *Bu zhi bu xing* (不知不行): Shancha and Cuiju

Shancha and Cuiju are both novice English teachers working in the same private secondary school at the time of this study. Shancha obtained a BA in English Teaching but said that she knew little about CRT since there were no specific courses for this in her study. Cuiju studied English Translation and confessed that her limited knowledge in English Teaching was from her preparation for the Teacher Certification Examination and some in-service teacher training activities in the school. Both teachers were in the “bu zhi bu xing” engagement regarding CRT. They had limited knowledge of CRT and failed to practice CRT in their teaching. Worthy of mentioning is that even though they were explained the concept of CRT by the researchers in the interviews, they still seemed reluctant to do it.

Concerning curriculum and instruction, Shancha did not make their teaching culturally relevant and believed that “not all students are interested in English or can learn it well” (Shancha-Interview). From her observation, some students had very poor English proficiency upon entry to junior secondary school, which made them less motivated in English learning. Even though some students were driven by extrinsic motivation, like achieving good marks in exams, they could not do well. Shancha said she could do a little with this as she could not lend extra help to all those stragglers in her class. Meanwhile, unlike urban families, few rural families could afford cram schools to further support their children in learning English. Acknowledging this as “a tough problem” (Shancha-Interview), Shancha said she did not know how to tackle it.

Cuiju failed to practice CRT in curriculum and instruction. As mentioned above, she did not receive formal teacher education before she started teaching and “wholly imitated her[my] mentor in terms of her[my] teaching” (Cuiju-Interview). While her mentor did not emphasize CRT in the lesson preparation or implement culturally responsive pedagogy in the teaching, Cuiju believed that it was the standard operating procedure (SOP) for her to learn and follow her mentor in teaching without any doubts.

Regarding classroom management, Shancha did not try to include every student due to her belief that “not all students are interested in English and can learn it well,” as mentioned previously. Meanwhile, she seemed to pay little attention to establishing a good learning atmosphere and good relationships with her students with the following considerations:

*It was the headteacher of the class instead of a subject teacher’s duty to establish a good learning atmosphere*…*Some experienced teachers told me not to be too close with my students since they were still children anyway and could not behave as rationally as adults.* (Shancha-Interview)

Cuiju also demonstrated little emphasis on CRT in classroom management and took the attitude of “let nature take its course” (Cuiju-Interview). In her teaching, Cuiju did not want to force students to engage in her teaching, given that some just wanted to keep a distance from their teachers.

Regarding student assessment, both teachers confessed that they used standardized tests as the primary instrument to assess students’ English learning. Shancha did so because she had to align with students’ parents’ expectations. Since parents sent their children to Shancha’s school (a private school) just to enhance their marks, teachers “ought to be practical and meet parents’ expectations first” (Shancha-Interview). Unlike Shancha, Cuiju emphasized standardized tests because “standardized tests create fairness for rural students” (Cuiju-Interview). According to Cuiju, due to the less favorable social-economic status, rural students had less access to seeing the outside world and thus had a limited scope of knowledge compared with urban students. Under such a circumstance, Cuiju thought what her students could compete for or earn the opportunities for better education was the grade of standardized tests, which did not require much extra-curricular knowledge.

Lastly, both teachers presented limited perspectives on cultural enrichment in their teaching. Before the study, Shancha did not know much about CRT. After the researchers explained to her, she said, “cultural enrichment does not belong to English teaching in rural schools…even if teachers try to do it in their teaching, students cannot benefit much since they can hardly understand” (Shancha-Interview). As elaborated by Shancha, cultural enrichment was the job for parents. However, since most rural parents could not/were not able to do this job satisfactorily, teachers could not do any better, and it was a waste of time talking about cultures in her English teaching. Cuiju was unaware of cultural enrichment before the interview, either. After the researchers explained the concept to her, she used the metaphor “textbook content is the dinner, while cultural enrichment is the snack” to describe her understanding (Cuiju-Interview), which indicated the periphery role of cultural enrichment in her teaching.

To summarize, this section displays Shancha’s and Cuiju’s engagement with CRT in their teaching. As revealed by the findings, both teachers did not acknowledge the significance of and practice CRT well in their teaching, influenced by internal factors like teachers’ beliefs (e.g., “not all students are interested in English and can learn it well”) and their professional identities (e.g., “It was the headteacher of the class instead of a subject teacher’s duty to establish a good learning atmosphere”), as well as external factors, including insufficient professional development training, parents’ expectation on the relatively expensive private schooling, rural students’ family background and rural education conditions.

### *Zhi xing he yi* (知行合一): Shaoyao and Qiangwei

Shaoyao and Qiangwei were experienced English teachers. Shaoyao had taught English in an urban private primary school and felt disparities after working in the rural secondary school. She said that the school environment was not satisfying and lacked advanced teaching equipment like personal computers. Moreover, most of her students were left-behind children whose English proficiency was remarkably lagged behind and whose parents were not involved much in their schooling, and some even held a biased understanding of English learning (i.e., learning English is useless for rural students).

Qiangwei’s first job was teaching English in an urban cram school for almost 6 years, which enabled her to develop an up-to-date educational philosophy and various English teaching pedagogies. Compared with her previous students at the cram school in the city, Qiangwei stated that her students in the rural school were poorer in English, and their parents were not actively engaged in their children’s schooling. At the time of this study, Shaoyao was teaching in a rural public secondary school, and Qiangwei worked in a rural private secondary school. Both teachers were teaching English to Grade 8 students. Regarding CRT, both teachers were in the “*zhi xing he yi*” type of engagement. That is, both teachers had a high level of cognitive, emotional, and social engagement. They were clear about CRT, highly acknowledged the significance of this concept, and proactively realized it in their teaching.

Within curriculum and instruction, Shaoyao and Qiangwei tried to practice CRT driven by their beliefs that “CRT cares much about students’ cultures and personal backgrounds” (Shaoyao-Interview) and “CRT should be inclusive and relaxing” (Qiangwei-Interview). In Shaoyao’s teaching, she tried to include those sensitive topics for rural students, such as parenting, parents’ marital status, and family bonds. In Shaoyao’s class, most students were left behind by their parents who went to big cities as migrant workers. These students lived with their grandparents and several even lived by themselves all year round. Thus, the topics about parents could be subtle to them. Shaoyao thought that teachers needed to learn about students’ family backgrounds. Therefore, when she met a new cohort of students, she tried to compile students’ profiles via different methods, including talking with students, making phone calls, paying home visits and so on, from which she developed a deeper understanding of every student. Such understanding, as reflected by Shaoyao, considerably “enhanced flexibility in my[her] English teaching” since she could “well handle sensitive topics that came up in the teaching content and avoid awkward situations” (Shaoyao-Interview). Apart from handling sensitive topics, Shaoyao also did well in designing her teaching to cater to students’ learning needs based on the needs analysis, like integrating familiar topics, playing more games, using on-site teaching resources, *etc*.

Qiangwei observed that some contents in the textbooks were too distant from her students’ life in rural China, which led to a sense of alienation and discouraged student engagement in the classroom. For example, one passage in the textbook talked about making a turkey dinner on Thanksgiving Day. According to Qiangwei’s experience, if the teacher tried to explain the passage line by line, “most students would be puzzled and quickly lost their concentration since they have little background knowledge about Thanksgiving Day” (Qiangwei-Interview). The same would happen to topics on science and technology, like space stations. Most of Qiangwei’s students did not care about such topics, as these were not the main content that would appear in *Zhongkao*. If they were just required to read, listen, and finish the questions, the teaching would be “meaningless and a waste of time” (Qiangwei-Interview). Thus, the teacher’s role was to “supplement, enrich, and consummate the teaching materials to reduce students’ sense of unfamiliarity” (Qiangwei-Interview). For example, when teaching the passage about Thanksgiving Day, after Qiangwei showed the class a video about the background as the supplement, students were excited and engaged in the passage learning.

*The classroom was quiet when the teacher played the video, and all the students watched carefully. After the video, they actively proposed some questions like “why did they go to the new place?”, “why didn’t they grow rice as we do?”. While some questions were obviously beyond Qiangwei’s preparation, this video obviously aroused students’ interest on Thanksgiving Day.* (Qiangwei-Classroom observation)

Qiangwei’s another purpose for supplementing teaching materials was to make her teaching more relaxing. As observed by Qiangwei, her students were interested in watching video clips, listening to music, and playing games. Qiangwei found that while participating in such activities, her students were relaxed and more engaged. Later, Qiangwei was happy to overhear that her students liked English the most among all the subjects, which greatly enhanced her confidence in realizing CRT.

Concerning classroom management, both teachers tried to make their teaching accessible to all of their students in the classroom and tap into the students’ cultural capital. Shaoyao believed that “teachers cannot give up any child” (Shaoyao-Interview). In her first class, Shaoyao randomly called a boy (Xiwang, pseudonym) to answer a question. All the other students burst into laughter since Xiwang had learning difficulties. The below classroom excerpt demonstrates how Shaoyao successfully handled the situation.

*Shaoyao: Xiwang, 我的问题是[My question is] “where did you go on vacation?”. 你知道是什么意思吗[Do you know what does it mean]?*
*Xiwang: (Shook his head)*
*Shaoyao: 意思是“你假期去了哪儿”[Meaning “where do you go on vacation”]?明白了吗[Are we clear]?*
*Xiwang: (Nodded his head)*
*Shaoyao: 那你暑假去了哪儿[Then where do you go on vacation]?*
*Xiwang: 在家[At home]。*
*Shaoyao: 在家怎么说 [How do you say “zaijia (at home)”]?*
*Xiwang: (Shook his head)*
*Shaoyao: 来跟老师说[Follow me]。 I stayed at home (while writing this sentence on the blackboard).*
*Xiwang: I stayed at home.*
*Shaoyao: I stayed at home (While pointing to the sentence on the blackboard).*
*Xiwang: I stayed at home.*
*Shaoyao: Where did you go on vacation? (Facing Xiwang)*
*Xiwang: I stayed at home.*
*Shaoyao: Good, 你答对啦!你们看, 他今天不会, 我们教了他之 后他不就会了嘛。, 你们今天不会没关系, 老师不会笑你, 所以别人不会你 也没有必要去笑 人家。 我们毕竟有很多知识, 你能保证你都知道吗, 对吧？[Good, you got it! He knew how to say it after we taught him, right? If you cannot do it, don’t worry. I won’t laugh at you. But if you can do it, please don’t laugh at those who cannot do it yet. It is not necessary. Knowledge is indefinite, and we cannot know everything, right?] (Facing all the students)*
*Students: 对[Yes]!*
*Shaoyao: Good! 那现在我们一起祝贺希望学习到了新知识[Good! Now we celebrate that Xiwang has learned new content in this unit]!*
*Students: (Clapped their hands)*
*Xiwang: (Sat straight and looked at Shaoyao with gratitude)*
(Shaoyao-Classroom Observation)

In the above excerpt, Shaoyao firstly tried to include the student Xiwang and ensure he could answer the question by teaching him several times. After the student gave the correct answer, Shaoyao seized the opportunity and taught students that they should be inclusive.

Like Shaoyao, Qiangwei also tried to create an inclusive atmosphere and made students feel “they are valuable in the class and the teacher cares about them” (Qiangwei-Interview). Some of Qiangwei’s students were not interested in English learning and kept silent in the classroom activities. Qiangwei never ignored them because of this but assigned them other activities like helping the teacher maintain discipline and collect assignments. In this way, Qiangwei successfully made these silent students feel included in her class: “they are still not active, but they can be good listeners now when others are speaking in class” (Qiangwei-Interview).

Regarding student assessment, both teachers did not rely solely on the standardized tests. Shaoyao believed that “students’ marks in the standardized tests are just auxiliary in assessing students’ learning” (Shaoyao-Interview), and this belief was formed by her growing understanding of her students. Through her interactions with students, Shaoyao learned that some students wanted her to know they were good children, despite their unsatisfying performance in English learning. In her teaching, Shaoyao tried to evaluate students’ learning by considering other variables, such as students’ participation in classroom activities, their personal traits, and attitudes toward English learning. Doing this was “obviously difficult due to the emphasis of *yi fen san lv* in rural education,” but Shaoyao wanted to insist because “it was helpful for the children in the long run” (Shaoyao-Interview). Like Shaoyao, Qiangwei also wanted to “be myself[herself] despite the test-oriented culture in my[her] school” (Qiangwei-Interview). In student assessment, Qiangwei integrated teacher-assessment, self-assessment, and peer-assessment. By doing this, “students’ voices were heard and respected. In the meantime, they could recognize themselves and their classmates, which is beneficial for students” (Qiangwei-Interview).

As for cultural enrichment, Shaoyao believed that “for rural students, building appropriate values is sometimes more important than studying the subject content” (Shaoyao-Interview). As observed by Shaoyao, many students in her school were left-behind children who lacked parental custody and support. Moreover, these students were usually the targets of the local school dropouts and under negative influences. Some students were addicted to phone games and often skipped classes; some boys even carried lighters and steel knives to school or were involved in minor crimes. Under such a situation, Shaoyao thought it was more pressing to help students build appropriate values and reduce their possibility of going astray. On the one hand, she seized every possible opportunity to educate her students. For example, in the unit about future careers, Shaoyao led students to discuss how to be a good citizen in the future. On the other hand, she acted as a role model, intending to influence her students “like the rain moistening the crops gently and silently (*run wu xi wu sheng*)” (Shaoyao-Interview). For instance, when the basketball team in her class provoked conflict with others, she proactively apologized on behalf of her students. She took the initiative to resolve the conflict, which set herself as a role model and became an integral part in the process of her students’ cultural conditioning. Some students felt remorse, cried, and became more polite since then.

Qiangwei also practiced cultural enrichment in her teaching based on her belief that “cultural awareness constituted a key and even the most important aspect of the core competence of English discipline” (Qiangwei-Interview). In Qiangwei’s understanding, the cultures embedded in English learning should include international and Chinese cultures in general and local and students’ cultures in particular. English teachers should guide students to navigate all these cultures intelligently, achieving the objective of “acknowledging and respecting the cultural differences, being proud of and maintaining students’ own cultures” (Qiangwei-Interview). In Qiangwei’s teaching, she formed an ICLY (International-Chinese-Local-Yours) cultural enrichment model. For example, after they learned the passage about Fuji Mountain in Japan, Qiangwei led students to talk about famous mountains in China, such as the Yellow Mountain and Mount Qomolangma; famous mountains in the local areas, such as Wudang Mountain and Shennong Peak; and the hills in their villages or around their houses. As reflected by Qiangwei, the FCLY model significantly ignited students’ interest in topics like these. In the meantime, it also promoted the students’ knowledge and affection for the world, their country, and their hometown.

Summarizing, this section shows Shaoyao’s and Qiangwei’s engagement with CRT. Both teachers clearly recognized the role of CRT and practiced it well in their teaching. Their beliefs in teaching, their professional identities and their pedagogical knowledge promoted their engagement with CRT in the rural schools. However, various external factors hindered the two teachers from more effectively practicing CRT, such as incomprehension from colleagues and the test-oriented culture. To create more cultural input in the teaching for her student, Shaoyao used to reading books in the field (i.e., Democracy and Education by John Dewey, 1923) at school. But she gave up doing so in front of her colleagues because of their sarcastic comments about her reading. Due to the uneven distribution of the educational resources, the students in the rural schools had poorer proficiency and thus made both teachers reduce time for cultural enrichment since her students were entering Grade 9, and *Zhongkao* was around the corner.

## Discussion

Adopting a multi-case design, we identified four types of engagement among eight EFL teachers and the internal and external factors influencing their engagement with CRT in their teaching contexts.

To begin with, previous studies treated the concept of teacher engagement as a united whole. They discovered different kinds of engagement, including incidental, ambivalent, integral (Jiang et al., 2021), disturbing, auxiliary, and integral (Zou et al., 2021). In contrast, our study looks into the different aspects of teacher engagement, i.e., cognitive, emotional, and social engagement (Klassen et al., 2013). Our findings suggest that an individual teacher could demonstrate different levels of engagement in the abovementioned three aspects. For example, Shuixian and Meigui realized CRT well in their work. Still, they knew little about CRT, thus demonstrating a high level of emotional and social engagement but a low level of cognitive engagement. By contrast, Dujuan and Fengxian knew the idea of CRT but were reluctant to realize it in their teaching due to some practical concerns, thus demonstrating a high level of cognitive engagement but a low level of emotional and social engagement. Considering such variations among the participants and underpinned by the Chinese concepts of “*zhi*” (which refers to knowledge) and “*xing*” (which denotes practice), we identified four types of engagement among the eight participants, i.e., *xing er bu zhi* (Shuixian and Meigui), *zhi er bu xing* (Dujuan and Fengxian), *bu zhi bu xing* (Shancha and Cuiju), and *zhi xing he yi* (Shaoyao and Qiangwei) (see Table 2).

The findings of this study reveal that “*zhi*” corresponds to the cognitive aspect of teacher engagement and “*xing*” relates to the social aspect of teacher engagement. Coupled with personal epistemology in teacher education (Brownlee and Berthelsen, 2008), Shuixian’s and Meigui’s engagement with CRT (*xing er bu zhi*) could be interpreted as practice informed by their tacit practical knowledge or implicit theories-in-use (Meijer et al., 2002). While both teachers practiced CRT well in this study, one possible risk of such engagement is that teachers might maintain the *status quo* for long if no opportunity is offered to actively interrogate their current teaching practices, develop their practical knowledge, and improve their teaching practices. Different from Shuixian and Meigui, the knowledge of Dujuan’s and Fengxian’s engagement with CRT (*zhi er bu xing*) was mainly composed of espoused theories, which teachers use to explicate their understandings of a particular concept or their behaviors (Argyris and Schon, 1974). One possible risk within such engagement is that espoused theories might be inapplicable or even misleading. For example, Dujuan and Fengxian thought CRT and academic success were incompatible. To better enhance their students’ performance in high-stake examinations like *Zhongkao*, they chose not to implement CRT. Such an understanding contradicts previous findings that academic success constitutes one of the essential tenets of CRT (Ladson-Billings, 2009; Gay, 2010). This also reminds us of Wang Yangming, a great Chinese philosopher in Ming Dynasty who argued that “*zhi*” (knowledge) in “*zhi er bu xing*” might not be genuine because it is based primarily on persons’ perceptions, but not on “*xing*” in authentic contexts, while “*zhi*” and “*xing*” are inseparable (Li, 2021). Lastly, Shaoyao’s and Qiangwei’s engagement with CRT (*zhi xing he yi*) could be seen as praxis, which is defined as “reflection and action upon the world in order to transform it” (Freire, 2000). Both teachers knew and practiced CRT well in their teaching, and this type of engagement is preferable in teacher education (Jackson and Boutte, 2018). While the “*zhi*” and “*xing*” explication of EFL teacher engagement with CRT emphasizes cognitive and social aspects of engagement, it does not overlook emotional engagement. On the contrary, our findings demonstrate that emotional engagement mediated the other two aspects of teacher engagement. As shown in Table 2, the eight EFL teachers’ emotional and social engagement levels were consistent. Teachers’ high emotional engagement led to high social engagement and vice versa. Such a finding testifies to the decisive role of teacher emotion in their teaching (Burić, 2019).

This study also identified internal and external factors influencing EFL teacher engagement with CRT. Internal factors include EFL teachers’ beliefs about English language education in rural areas, their knowledge about CRT, and their commitment to their careers in rural schools. External factors include the test-oriented culture in rural schools, the teacher evaluation regime (i.e., *yi fen san lv*), and social conditioning of the students (e.g., the parents, schools, and other colleagues). While these factors are not new and have been repeatedly reported in previous studies (e.g., Nieto, 2010; Bao et al., 2021; Jiang et al., 2021; Wu et al., 2022), they represented an unsatisfying environment for rural EFL teachers to conduct CRT in China with at least four outstanding problems. Firstly, rural EFL teachers lacked professional learning opportunities. Thus, some concepts like CRT remained implicit (i.e., Shuixian and Meigui) or even unknown (i.e., Shancha and Cuiju) among rural EFL teachers, especially novice teachers. Secondly, some rural EFL teachers lacked commitment to rural education and were less motivated to conduct CRT. For example, Meigui did not plan to stay in the rural school and the teaching profession for long. While she generally did an excellent job conducting CRT, she did little in cultural enrichment. She regarded herself as a visitor and was not interested in learning and integrating local cultures in her EFL teaching. Thirdly, rural EFL teachers were trapped by the test-oriented culture and the associated teacher evaluation regime (e.g., *yi fen san lv*), which discouraged them from implementing CRT in their teaching. Although some teachers (e.g., Dujuan) acknowledged the significance of CRT in English teaching, they would not waste the class time for the students’ *Zhongkao* preparation. Lastly, rural EFL teachers were negatively influenced by different stakeholders in rural schools. For example, the students’ low English proficiency made teachers (e.g., Fengxian) hardly cover cultures in their teaching; the parents’ emphasis on marks forced teachers (e.g., Shancha) to solely focus on standardized tests; and colleagues’ sarcastic comments made some teachers (e.g., Shaoyao) flinch to read and learn more about CRT practices.

Despite these negative influencing factors, the findings also suggest the significance of rural EFL teachers’ agentive and reflective stances in engaging with CRT (Jackson and Boutte, 2018). As indicated by the results, Shaoyao and Qiangwei insisted on practicing CRT within the unsatisfying rural EFL education environment. Specifically, Shaoyao believed that “for rural students, building appropriate values is sometimes more important than studying the subject content” and “it was helpful for the children in the long run”. Qiangwei wanted to “be herself” despite the test-oriented culture in the rural school. She even developed an ICLY model for cultural enrichment, which further benefited her EFL teaching. By contrast, Shancha and Cuiju knew little about CRT and were reluctant to explore it even after the researchers explained the concept. In their teaching, Shancha did not intend to include every student and regarded this as “a tough problem.” Cuiju wholly and even blindly imitated her mentor’s teaching without any doubt. The lack of agency in the two teachers led to their unsatisfying performance in CRT.

## Implications and conclusion

This study explores EFL teacher engagement with CRT in rural schools in China. By identifying four types of teacher engagement and the specific internal and external influencing factors, this study adds insightful understanding to the underexplored topic of language teachers’ engagement with CRT, provides first-hand information on the professional lives of rural EFL teachers, and highlights various problems of English Language education in rural China.

Several implications can be generated to enhance rural EFL teacher engagement with CRT in China and similar contexts worldwide. To start with, for EFL teacher educators, given that CRT can benefit both teaching and learning (Gay, 2010, 2018) but remains a strange concept to some rural EFL teachers, particular courses in pre-service teacher education and workshops in in-service teacher training can be provided to rural EFL teachers so that they can develop a theoretical understanding of CRT and devise strategies for implementing CRT in their teaching. Secondly, for the administrators in rural schools, it is highly appreciated if they can improve the evaluation regime for teachers (i.e., yi fen san lv), which has been driven by students’ marks in high-stake tests. It then can create time and space for the teachers to make their class more inclusive and engaging. Meanwhile, a collegial office is a necessity to promote teachers’ enthusiasm for engaging and exploring CRT (e.g., Shaoyao’s case) in a supportive, inclusive and positive professional environment. Besides, rural teachers’ agentive and reflective stances are essential in engaging with CRT (e.g., Qiangwei’s case). Thus, despite the possible negative influences from the under-resourced context, rural EFL teachers should be encouraged to learn and embrace CRT, follow their original beliefs about education, and try to be reflective in their work. In this way, the teachers could provide their students with more informative, inclusive and engaging content in class and help them build values, create visions, and develop skills for the future.

While rich insights have been generated in this study, the findings need to be interpreted in light of the following limitations. First, this study relied on participants’ self-report data. Due to the COVID-19 pandemic and the strict preventive measures of the research schools, we were not allowed to enter the campus for an extended period, and our original data collection plan was thus interrupted. Specifically, we only observed one period for each teacher. Therefore, future research requires more observations of rural EFL teachers’ practices, student evaluations of teacher engagement, and administrator perspectives. In addition, this study adopted a qualitative research design and focused on eight rural EFL teachers. Although this is an appropriate way to generate a thick description to explore EFL teacher engagement with CRT in rural schools, future researchers may want to conduct empirical work with quantitative data that may provide further evidence.

## Data availability statement

The original contributions presented in this study are included in the article/supplementary material, further inquiries can be directed to the corresponding author/s.

## Ethics statement

The studies involving human participants were reviewed and approved by School of Foreign Languages, Huazhong University of Science and Technology. The patients/participants provided their written informed consent to participate in this study.

## Author contributions

DK was mainly responsible for the research design, data collection, data analysis, and manuscript drafting. MZ was primarily responsible for the research design, data analysis, and part of the manuscript drafting. JC was responsible for the research design, participant recruitment, data analysis, and part of the manuscript drafting. All authors contributed to the article and approved the submitted version.

## Footnotes

1 In China, *jiaoshi bianzhi* refers to the authorized number of personnel in public schools. The government will be responsible for these teachers regarding their health care, salary, allowance, *etc*.

2 In China, the senior high school entrance examination (*Zhongkao*) is a critical examination in mainland China throughout which junior secondary school students will be placed into different kinds of education institutions.
